# URM1-Mediated Ubiquitin-Like Modification Is Required for Oxidative Stress Adaptation During Infection of the Rice Blast Fungus

**DOI:** 10.3389/fmicb.2019.02039

**Published:** 2019-09-10

**Authors:** Luyang Wang, Xuan Cai, Junjie Xing, Caiyun Liu, Ahmed Hendy, Xiao-Lin Chen

**Affiliations:** ^1^The Provincial Key Lab of Plant Pathology of Hubei Province, College of Plant Science and Technology, Huazhong Agricultural University, Wuhan, China; ^2^State Key Laboratory of Hybrid Rice, Hunan Hybrid Rice Research Center, Changsha, China; ^3^Department of Agricultural Botany, Faculty of Agriculture (Saba Basha), Alexandria University, Alexandria, Egypt

**Keywords:** ubiquitin-like modification, urmylation, infection process, oxidative stress, *Magnaporthe oryzae*

## Abstract

Ubiquitin is a small modifier protein which is usually conjugated to substrate proteins for degradation. In recent years, a number of ubiquitin-like proteins have been identified; however, their roles in eukaryotes are largely unknown. Here, we describe a ubiquitin-like protein URM1, and found it plays important roles in the development and infection process of the rice blast fungus, *Magnaporthe oryzae*. Targeted deletion of *URM1* in *M. oryzae* resulted in slight reduction in vegetative growth and significant decrease in conidiation. More importantly, the Δ*urm1* mutant also showed evident reduction in virulence to host plants. Infection process observation demonstrated that the mutant was arrested in invasive growth and resulted in accumulation of massive host reactive oxygen species (ROS). Further, we found the Δ*urm1* mutant was sensitive to the cell wall disturbing reagents, thiol oxidizing agent diamide and rapamycin. We also showed that URM1-mediated modification was responsive to oxidative stresses, and the thioredoxin peroxidase Ahp1 was one of the important urmylation targets. These results suggested that URM1-mediated urmylation plays important roles in detoxification of host oxidative stress to facilitate invasive growth in *M. oryzae*.

## Introduction

Post-translational modification (PTM) of proteins to regulate their functions is an emerging theme. Ubiquitination is one of the most widely existing PTMs in eukaryotes and is involved in regulation of numerous cellular processes. Frequently, ubiquitination usually covalently attaches ubiquitin, a 76-residue protein, to target proteins for degradation, but other regulatory functions of ubiquitination are also found (Schnell and Hicke, 2003; Komander and Rape, 2012). Interestingly, besides ubiquitin, a number of ubiquitin-like proteins (Ubls) are also found to be present in eukaryotes, including SUMO1, SUMO2, SUMO3, NEDD8, ISG15, FAT10, UFM1, ATG8, ATG12, HUB1, and URM1 (Hochstrasser, 2000; van der Veen and Ploegh, 2012). SUMO proteins usually modify target proteins to alter their localization, stability, and interaction with other proteins, therefore mediating transcriptional regulation, chromatin remodeling, cell cycle progression, and DNA repair (Geiss-Friedlander and Melchior, 2007). The best-characterized NEDD8 modified proteins are members of the cullin family, components of the cullin-RING E3 ubiquitin ligase (Hochstrasser, 2009). Atg8 and Atg12 are involved in conjugation systems during autophagy (Hochstrasser, 2009). Similar to ubiquitin attachment, the Ubls also recruit a series of enzymes, including E1 (activating enzyme), E2 (conjugating enzyme), and E3 (protein ligase), to facilitate modification. Through the function of these enzymes, the Ubls can be attached to the lysine residues of the target proteins through their C terminus (Haas and Siepmann, 1997).

Different from ubiquitination, these Ubls-mediated modifications are not usually used for protein degradation but are used for regulating the function or localization of the target proteins, and thus are involved in the regulation of many cellular processes (Lammer et al., 1998; Liakopoulos et al., 1998; Mahajan et al., 1998; Kawakami et al., 2001). For example, small ubiquitin-related modifier (SUMO) modification is a well-characterized Ubl modification. Up to now, thousands of the SUMO targets have been identified in different eukaryotic cells, including Ran GTPase-activating protein RanGAP1 (Mahajan et al., 1998), inflammatory-response regulatory protein IκBα (10), and the septin ring components in *Saccharomyces cerevisiae* and *Magnaporthe oryzae* (Desterro et al., 1998; Liu et al., 2018). NEDD8 is another Ubl protein, which is the most similar to ubiquitin in sequence. NEDD8 can target Cullin proteins (scaffold proteins for the assembly of RING E3 ligases) to promote ubiquitination and proteasomal degradation (Rabut and Peter, 2008).

Compared with SUMO and NEDD8, URM1 (Ubiquitin Related Modifier 1), which was firstly identified and studied in *S. cerevisiae* (Goehring et al., 2003a, b; Pedrioli et al., 2008; Leidel et al., 2009), was relatively less studied. In the URM1-mediated urmylation process, the activation enzyme E1 Uba4p is the only found component of the conjugation pathway, while the E2 and E3 have not been identified. URM1p forms a thioester bond to interact with Uba4p (Furukawa et al., 2000). In *S. cerevisiae*, by the function of Uba4p, URM1 can be covalently conjugated to a thioredoxin peroxidase protein Ahp1p for responding to oxidative stress (Goehring et al., 2003a). Recently, several other targets of URM1, including MOCS3, ATPBD3 and CTU2, as well as the nucleocytoplasmic shuttling factor cellular apoptosis susceptibility protein, have also been found in mammalian cells, through which URM1 can target different pathways upon oxidant treatment (van der Veen et al., 2011). The sulfurtransferase MOCS3 is involved in a protein involved in molybdenum cofactor biosynthesis (Marelja et al., 2008). The thiouridylases ATPBD3 and CTU2 can mediate tRNA thiolation of wobble uridines (Huang et al., 2008; Schlieker et al., 2008). Recent studies demonstrated URM1 also functions as a sulfur carrier to regulate thiolation of cytoplasmic tRNAs (Björk et al., 2007; Schlieker et al., 2008; Leidel et al., 2009).

In *S. cerevisiae*, disruption of *URM1* led to reduction of growth, increased sensitivity to temperature and rapamycin (Furukawa et al., 2000), and defects in agar invasive growth under starvation (Goehring et al., 2003b). The disruption mutant of *URM1* is also decreased in resistance to calcofluor white (CFW) and diamide (Fichtner et al., 2003; Goehring et al., 2003a). The URM1-mediated urmylation can help yeast strains to grow at high temperatures by stabilizing the tRNA through thiolation (Sinha et al., 2008). Disruption of yeast *URM1* also led to the strains being sensitive to rapamycin and mis-localization of TOR (target of rapamycin) pathway downstream kinases gln3p and gat1p (Rubio-Texeira, 2007), indicating urmylation plays roles in the TOR signaling pathway. However, the function of urmylation is still largely unknown in other eukaryotes.

*Magnaporthe oryzae* is a fungal pathogen which causes rice blast disease, the most destructive rice disease worldwide. During infection, *M. oryzae* can develop an infection structure called appressorium to penetrate the host cells (Wilson and Talbot, 2009). During fungal penetration, the host cells usually activate a strong defense response (Jones and Dangl, 2006). To facilitate colonization in host cells, *M. oryzae* has developed different strategies to overcome the host defense response (Samalova et al., 2014). The oxidative stresses usually appear during early stages in the plant upon pathogen infection, which can be produced by reactive oxygen species (ROS) and thiol compound (Grene, 2002). ROS composed of the singlet oxygen (^1^O_2_), superoxide (O_2_^–^), hydrogen peroxide (H_2_O_2_) and hydroxyl radical (HO) can act as a sensor to regulate global patterns of gene expression in the defense process (Tripathy and Oelmüller, 2012). Thiol redox is partially regulated by the redox state of the glutathione pool (GSH/GSSG) (Tripathy and Oelmüller, 2012). During *M. oryzae* infection, a weak and temporary ROS burst occurs in susceptible rice, while in resistant response, a strong and sustained ROS burst is induced in resistant rice (Parker et al., 2009). Fungal pathogens have also developed strategies to counteract plant ROS stress, and several genes involved in ROS detoxification have been characterized in *M. oryzae* (Chi et al., 2009; Guo et al., 2010, 2011; Huang et al., 2011). In this study, we identified a Ubl gene *URM1* in *M. oryzae*. Functional analysis to *URM1* revealed urmylation is involved in colony growth, conidiation, and invasive growth in the host cells. URM1 plays an important role in detoxifying host oxidative stress, and it can modify thioredoxin peroxidase Ahp1.

## Materials and Methods

### Strains and Culture Conditions

*Magnaporthe oryzae* strain P131 served as the wild type strain in this study, and all the fungal strains (Supplementary Table S1) were cultured at 28°C on oatmeal tomato agar (OTA) plates. Genomic DNA and total RNA were extracted from mycelia cultured in liquid CM medium cultures (180 rpm) incubated at 28°C for 36 h. Colony diameters of the OTA plate colonies were measured at 120 hpi (28°C). Conidiation was examined by harvesting conidia from 7-day-old colonies cultured on OTA plates at 28°C under continuous light condition. For cell wall integrity assay, strains were cultured on CM plates added with 0.2 mg/ml Congo Red (CR) (Sigma-Aldrich, United States), 0.1 mg/ml CFW (Sigma-Aldrich, United States), and 0.005% Sodium dodecyl sulfate (SDS), and the colony diameters were measured after 5 days of growth. For oxidative stress sensitivity assay, strains were cultured on the CM plates supplemented with 10 mM H_2_O_2_, 1.5 mM diamide and 25 ng/ml rapamycin (Sigma-Aldrich, United States). To observe cell lengths, the mycelium was stained with 10 μg/ml CFW for 10 min in the dark and were observed under a fluorescence microscope (Nikon Ni90 microscope, Japan).

### Phylogenetic Tree Analysis and Protein Alignment

We use the protein sequence of MoURM1 as a query to search homolog proteins of different species through BLAST (basic local alignment search tool) on Enzembl Fungi website^1^. Clustal_W was used to align the amino acid sequences of homologous proteins in different species (Larkin et al., 2007). A phylogenetic tree was conducted in MEGA7, and the percentage of replicate in which associated taxa clustered together in the bootstrap test (1000 replicates) is shown next to the branches (Kumar et al., 2016).

### Gene Disruption and Complementation

Gene disruption was performed through a Split-PCR strategy as previously described (Goswami, 2012). For complementation, a fragment containing 1.5 kb promoter region, the *URM1* coding region and 0.5 kb terminator region was amplified and then cloned into the plasmid pKN (Supplementary Table S2; Chen et al., 2014). The complementation vector was transformed into the Δ*urm1* mutant. The CM plates were supplemented with 250 μg/ml hygromycin B (Roche, United States) for selecting the deletion transformants and 400 μg/ml neomycin (Amresco, United States) for selecting the complementation transformants.

### Subcellular Localization

To construct the vector for subcellular localization, the *URM1* gene was amplified and ligated into the C-terminal of vector GFP gene in pKNRG, which contains the constitutive promoter RP27 (Supplementary Table S2; Liu et al., 2018). The subsequent vector pKNRG-*URM1* was transformed into the Δ*urm1* mutant, and the transformants were selected by using 400 mg/ml neomycin. Transformants at different developmental stages and infection processes were used to observe subcellular localization under a confocal microscope Leica TCS SP8 (Leica Microsystems, Germany). All primers used in this study are summarized in Supplementary Table S3.

### Infection Assay

One-month-old rice seedlings (*Oryza sativa* cv. LTH) and one-week-old barley leaves (*Hordeum vulgare* cv. E9) were used for virulence tests. The conidia suspension with a concentration of 5 × 10^4^ conidia/ml in 0.025% Tween 20 was used to spray plants, then the plants were incubated in full humidity conditions at 28°C. Five days later, the disease lesion was examined.

The appressorium formation was tested by dropping the conidial suspension (1 × 10^5^ conidia/ml) onto a hydrophobic coverslip, and then the coverslip was incubated in a dark moist chamber at 28°C. The appressoria formation ratio was observed at 12 hpi under a microscope (Nikon Ni90, Japan). The infection process in host cells was tested by inoculating the conidial suspension (1 × 10^5^ conidia/ml) onto the lower barley leaves and incubating them in a dark moist chamber at 28°C. Infection processes were observed under a Nikon Ni90 microscope (Japan) by removing the fungus-infected lower barley epidermis at 24 and 30 hpi.

### DAB Staining Assay

Host-derived ROS was detected by staining with DAB (3, 3-diaminobenzidine, Sigma-Aldrich, United States) as described by Chen et al. (2014). Barley leaves were inoculated with the mutant and wild-type strains by dropping with the conidial suspension (1 × 10^5^ conidia/ml). At 30 hpi, the barley leaves were immersed in DAB solution (1 mg/mL, pH 3.8) at room temperature for 8 h, and then destained with clearing solution (ethanol:acetic acid = 94:4, v/v) for 1 h.

### Quantitative Reverse Transcription PCR Analysis

To evaluate the expression level of *URM1* at different developmental stages, different tissues were harvested as previously described (Liu et al., 2018). For the extraction of RNAs, samples of the appressoria were harvested at 3, 6, and 12 hpi on a hydrophobic plastic surface after spray inoculation with conidia suspensions of 5 × 10^5^ conidia/mL, and samples of the infection hyphae (IH) were harvested at 18, 24 and 42 hpi by collecting the barley epidermis after spray inoculation with conidia suspensions of 5 × 10^5^ conidia/mL. Then, the total RNA extracted from these samples was used for preparing the cDNA templates. The qRT-PCR was performed by using an SYBR Green PCR Master Mix kit (Takara, Dalian, China) on an ABI 7500 real-time PCR system (Applied Biosystems, United States). The expression level of each gene was normalized by *M. oryzae* β-tubulin (*MGG_03982*) (Che Omar et al., 2016), and then the expression level of URM1 in mycelium was set as 1.

### Western Blotting

For determination of the urmylation level, the *URM1:3* × *Flag* construct was transformed into the wild-type strain. For determination of Ahp1 urmylation, the *AHP1:3* × *Flag* construct was transformed into the wild type or Δ*urm1* mutant. Total proteins of different transformants were extracted and were used for Western blot analysis with anti-flag (1:5000, Abmart, China) antibody. To determine the effect of oxidative stresses on urmylation, mycelia of these transformants were treated with 0.5 mM diamide or 2 mM H_2_O_2_ for different times before protein extraction.

## Results

### Identification of URM1 in *M. oryzae*

**FIGURE 1:**
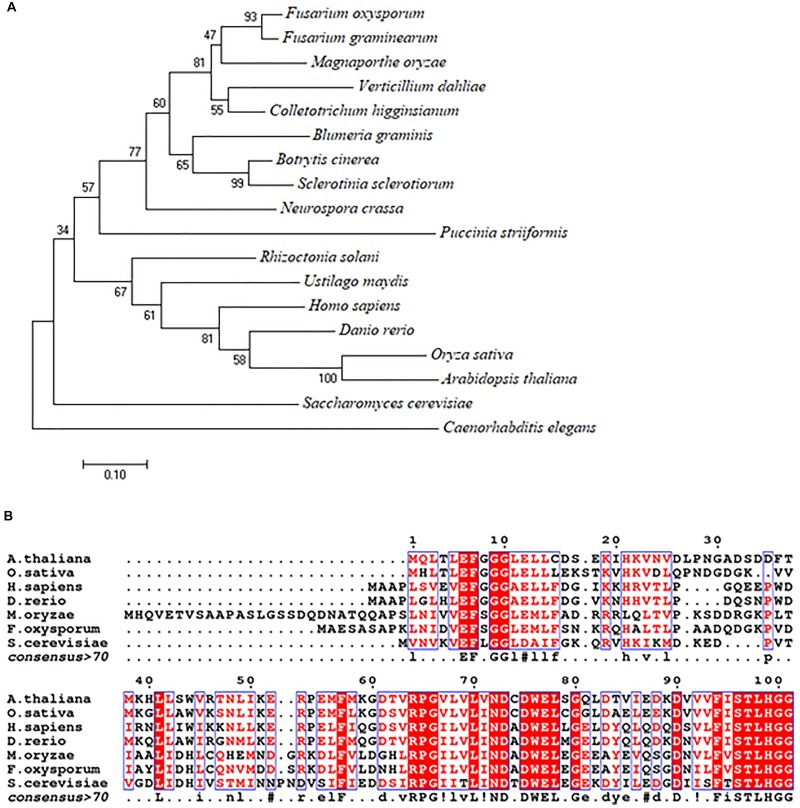
Phylogenetic tree and alignment analyses of the URM1 proteins. **(A)** A phylogenetic tree of the URM1 amino acid sequences in different species. Numbers at nodes indicate the bootstrap values. Scale bar indicates the number of amino acid differences per site. **(B)** Alignment of the amino acid sequences of URM1 from *M. oryzae* and other selected model organisms. Amino acid sequences were aligned with ClustalW (http://www.ch.embnet.org/software/ClustalW.html). Identical residues are colored by red regions, and similar residues are colored by red characters.

Using the *S. cerevisiae* URM1 sequence as a reference, we identified the *M. oryzae* URM1 (MGG_03978) via a search of the *M. oryzae* genome database (Ensembl Fungi). The *MoURM1* is predicted to encode a protein of 129 amino acids residues. Phylogenetic tree analysis was performed by using MEGA7 software, which demonstrated that the URM1 protein is closely matched to all tested ascomycete fungi, but not closely matched to other eukaryotes, including the basidiomycete fungi *Rhizoctonia solani*, *Ustilago maydis* and *Puccinia striiformis*. Among the analyzed organisms, *Fusarium oxysporum* (EWY_99348.1) and *Fusarium graminearum* URM1 are the closest matches to MoURM1 (Figure 1A). Multiple sequence alignment showed URM1 shares high homology to a number of proteins in different eukaryotes, including in *S. cerevisiae*, *F. oxysporium*, *Arabidopsis thaliana*, *Oryzae sativa*, *Danio rerio*, and *Homo sapiens* (Figure 1B).

### Expression Patterns and Targeted Gene Disruption of *URM1* in *M. oryzae*

To examine the gene expression changes of *URM1* at different developmental stages in *M. oryzae*, we extracted RNA from mycelia, conidia, germinated conidia, appressoria and host intracellular IH at 18, 24 and 42 hours post inoculation (hpi). The qRT-PCR analysis was used to identify the expression patterns. We calculated the expression level of each gene by using *M. oryzae* β-tubulin (*MGG_03982*) as a reference gene (Che Omar et al., 2016). The expression level of *URM1* reached its maximum peaks during formation of the appressorium and early IH formation stages, indicating important roles for *URM1* in these stages (Supplementary Figure S1).

To study the function of *URM1* in *M. oryzae*, the gene replacement construct was amplified by a split-PCR strategy (Supplementary Figure S2A) and then transformed into the protoplasts of the wild-type strain P131. The resulting transformants were screened by PCR and putative *URM1* gene deletion mutants were confirmed by RT-PCR (Supplementary Figures S2B,C). As a result, two independent Δ*urm1* deletion mutants with similar phenotypes were obtained. We randomly chose one mutant, KO1, for further analysis. The complement strains were also generated by transforming the native promoter-driven *URM1* construct into the deletion mutant.

**FIGURE 2:**
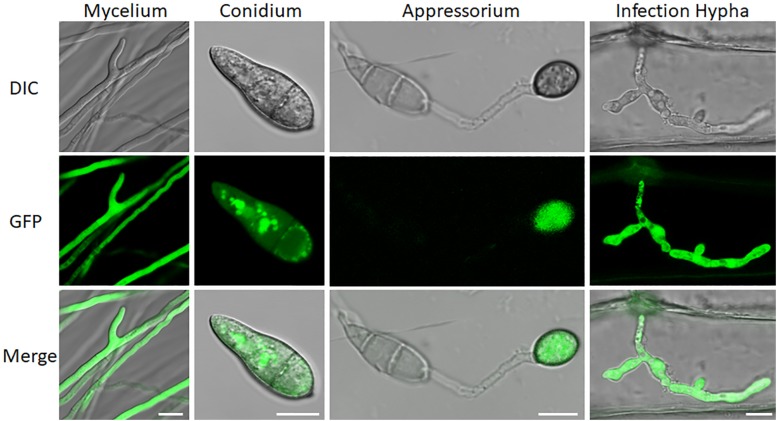
Subcellular localization of URM1 at different developmental stages of *M. oryzae*. Bar, 10 μm.

We also examined subcellular localization of a GFP-URM1 fusion protein at different developmental stages of *M. oryzae*. The GFP-URM1 fusion construct was introduced into the KO1 mutant and the subsequent transformants were verified for normal growth, conidiation, and infection and considered as complemented strains. One of which, cURM1, was examined under an epifluorescence microscope, the GFP signal of GFP-URM1 was detected in the cytoplasm of all tested tissues, including the hyphae, conidia, appressoria and IH (Figure 2). Interestingly, in conidia, GFP-URM1 was also detected in some granular structures (Figure 2).

### Roles of URM1 in Morphological Development

**FIGURE 3:**
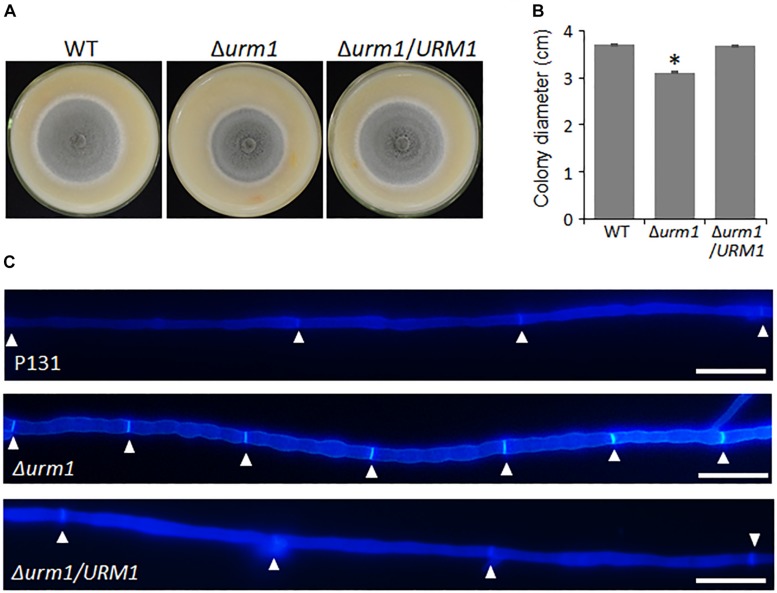
*URM1* is involved in vegetative growth. **(A)** Colony morphology of the wild type P131, Δ*urm1* mutant and complemented strains were observed on oatmeal tomato agar (OTA) plates at 28°C for 5 days. **(B)** The colony diameters were measured and subjected to statistical analysis. Error bars represent standard deviation and asterisk represents significant difference (*P* < 0.05). **(C)** Cell length of the hyphae tips in the wild type P131, Δ*urm1* mutant and complemented strains. The triangles indicate septa between cells.

**FIGURE 4:**
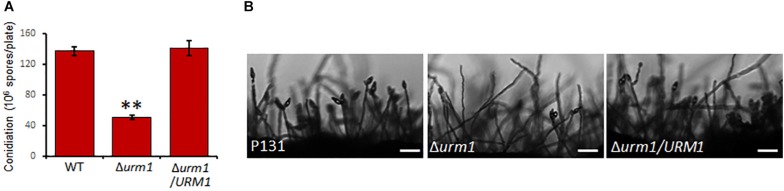
Disruption of *URM1* shows reduced conidial production. **(A)** Statistical analysis of conidiation of the wild type, Δ*urm1* mutant and complemented strains. Error bars represent the standard deviation and asterisks represent significant difference among strains (*P* < 0.01). **(B)** Conidial and conidiophore formation were observed under a light microscope. The indicated strains were grown on OTA plates for 5 days. Bar, 50 μm.

To investigate the roles of *URM1* in growth and development of *M. oryzae*, the Δ*urm1* mutant was cultured on OTA plate for 120 h at 28°C. In comparison with the wild-type P131, the Δ*urm1* mutant showed slight growth reduction (Figures 3A–C). This result indicated that *URM1* has a role in vegetative growth. We next investigated the role of *URM1* in conidiation. Conidia formation in the Δ*urm1* mutant on OTA plate was evidently reduced only around 36.5% compared to that of wild-type P131 (Figure 4A). Microscopic observation found that, although the number of conidiophores was normally formed, sparse conidia were formed on the conidiophores of the Δ*urm1* mutant, while dense conidia were formed on the conidiophores of the wild-type and complement strains (Figure 4B). These results suggested that *URM1* plays important roles in growth and conidia development.

**FIGURE 5:**
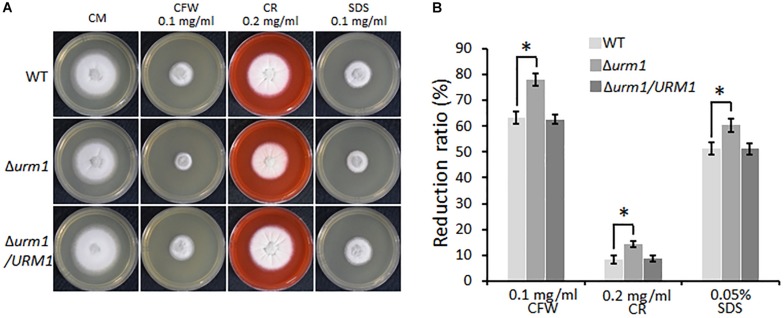
Deletion mutants of *URM1* are sensitive to cell wall disturbing agents. **(A)** Colony morphology of the wild type, Δ*urm1* mutant and complemented strains on CM plates supplemented with different cell wall disturbing agents. The colonies were photographed at 5 dpi. **(B)** Statistical analysis of growth reduction rates of colony growth under cell wall disturbing agents. Means and standard errors were calculated from three independent replicates. Significant differences were indicated by asterisks (*P* < 0.05).

We also tested the effect of *URM1* disruption on cell wall integrity of *M. oryzae*. Mycelial plugs were cultured on CM agar, respectively, supplemented with different cell wall perturbing reagents, including 0.1 mg/ml CFW, 0.2 mg/ml CR and 0.005% SDS. Compared with that of the wild-type strain, the Δ*urm1* mutant exhibited increased sensitivity to these reagents (Figure 5). These data indicated that *URM1* is required for cell wall integrity.

### URM1 Is Required for Full Virulence of *M. oryzae*

**FIGURE 6:**
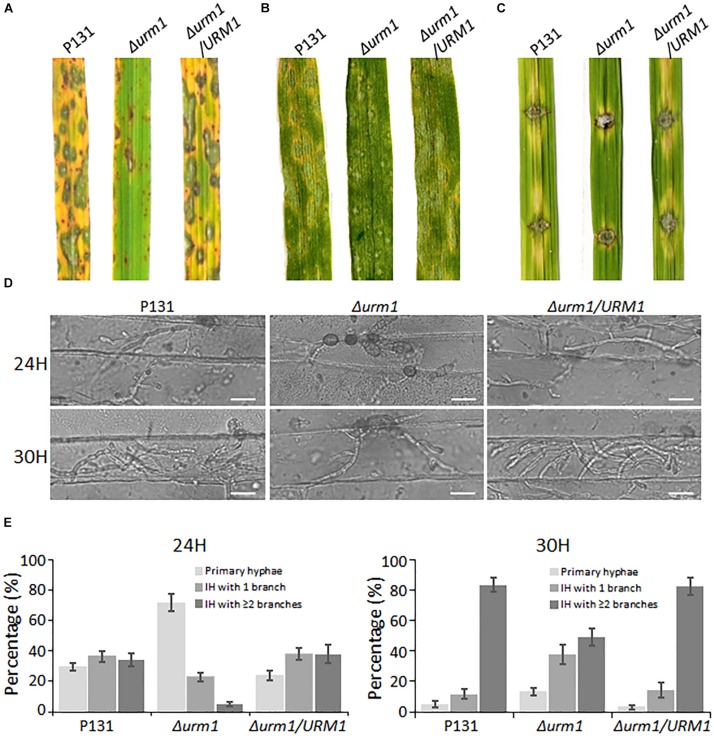
The Δ*urm1* mutant showed attenuated pathogenicity. **(A)** Virulence test on rice seedlings. The rice cultivar is *Oryza sativa* cv. LTH. **(B)** Virulence test on barley leaves. The barley cultivar is *Hordeum vulgare* cv. E9. Conidia suspensions (1 × 10^5^ conidia/ml) for each strain were sprayed on one-month-old rice seedlings and 7-day-old barley leaves. **(C)** Virulence test on wounded rice leaves. Rice leaves were slightly wounded by a needle before inoculation with a mycelial block of the wild type, Δ*urm1* mutant or complemented strain. Typical leaves were photographed at 5 dpi. **(D)** Colonization of the Δ*urm1* mutant on barley epidermis at 24 and 30 hpi. Bar, 25 μm. **(E)** Statistical analysis for different types of the infection hyphae (IH) at 24 and 30 hpi. Means and standard errors were calculated from three independent replicates (*n* > 100).

To determine whether *URM1* is required for pathogenicity, conidial suspensions of the Δ*urm1* mutant, wild-type and complemented strains were, respectively, sprayed onto one-month-old rice seedlings (*Oryzae sativa* cv. LTH). Tiny restricted lesions were found in Δ*urm1* mutant infected rice leaves, while the wild type and complementation strains caused numerous typical spreading lesions (Figure 6A). When the conidial suspensions of the above strains were sprayed onto one-week barley leaves, a similar result could be also observed (Figure 6B). These results indicated the deletion of *URM1* attenuated the virulence to rice and barley. In order to determine whether deletion of *URM1* affected *Magnaporthe* spread on wounded rice leaves, the mycelial agar plugs of different strains were inoculated onto the wounded rice leaves. At 5 days post inoculation (dpi), the lesions formed by the Δ*urm1* mutant spread much slower than the wild-type and complemented strains (Figure 6C), suggesting host intracellular colonization of the mutant was blocked.

To further elucidate the mechanism underlying the attenuated virulence in the Δ*urm1* mutant, we observed the cellular infection processes. We found that the appressorium formation of the Δ*urm1* mutant exhibited no difference with the wild-type strain (Supplementary Figure S3), indicating *URM1* could be not required for appressorium formation. However, when observing the invasive growth on barley epidermis at 24 and 30 hpi, we found the IH formation of the Δ*urm1* mutant was evidently slower than that of the wild-type and the complemented strains. At 24 hpi, more than 70% appressoria of the wild-type and complemented strains developed branched IH, while only around 30% appressoria of the Δ*urm1* mutant formed branched IH. At 30 hpi, more than 80% of the wild-type IH formed multiple branched IH, whereas it was no more than 50% in the Δ*urm1* mutant (Figures 6D,E). Taken together, *URM1* is required for invasive growth in the barley host cells during infection.

### Deletion of *URM1* Induced Accumulation of Host ROS

**FIGURE 7:**
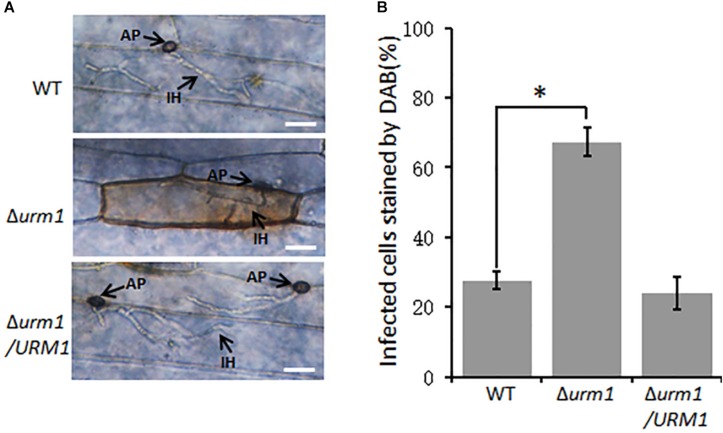
DAB staining assays of the wild type, Δ*urm1* mutant and complemented strains penetrated barley epidermis (*Hordeum vulgare* cv. E9). **(A)** DAB sraining assay. Barley epidermis was stained with DAB at 30 hpi. AP, appressoria; IH, infection hyphae. Bar, 20 μm. **(B)** Percentage of the infected cells stained by DAB. Significant differences were indicated by asterisks (*P* < 0.01).

Considering the Δ*urm1* mutant was arrested in invasive growth, we wondered if the mutant could induce host ROS accumulation. To test this possibility, the *M. oryzae* infected barley epidermis cells at 30 hpi were stained with 3, 3-diaminobenzidine (DAB) to detect ROS accumulation. As shown in Figure 7, around 70% of the Δ*urm1* mutant infected barley epidermis cells were detected with abundant reddish brown precipitates, while no more than 30% of the wild-type strain infected host cells could be stained, indicating the ROS reaction was indeed induced by the mutant in the host cells.

### Sensitivities of Δ*urm1* and Δ*ahp1* to Oxidative Stresses and Rapamycin

**FIGURE 8:**
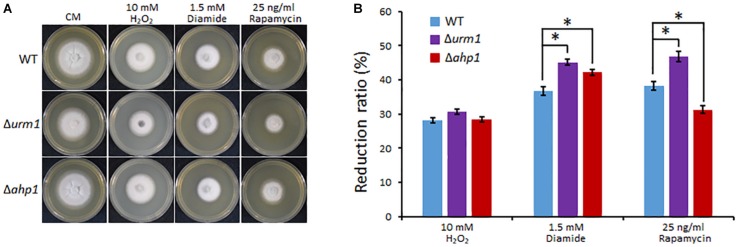
Deletion mutant of *URM1* is sensitive to H_2_O_2_, diamide and rapamycin. **(A)** The wild type, Δ*urm1* mutant and complemented strains were inoculated on CM plates supplemented with H_2_O_2_, diamide and rapamycin in indicated concentrations. The colonies were photographed at 5 dpi. **(B)** Statistical analysis of inhibition rate based on colony diameters. Means and standard errors were calculated from three independent replicates. Significant differences are indicated by asterisks (*P* < 0.05).

In *S. cerevisiae* and mammals, the urmylation pathway was found to be involved in oxidative-stress response, partially by regulating a thioredoxin Ahp1 (Goehring et al., 2003a; van der Veen et al., 2011). By using the yeast Ahp1 as a query, we identified the *M. oryzae* Ahp1 protein (MGG_00860) through BLAST search to the *M. oryzae* genome. We therefore obtained the *M. oryzae AHP1* deletion mutant and tested whether the deletion of *URM1* or *AHP1* resulted in sensitivity to oxidative stresses. The Δ*urm1* mutant seemed slightly sensitive to the H_2_O_2_ stress; however, the Δ*ahp1* mutant was not. Because *S. cerevisiae* Ahp1 may function as an antioxidant specific for the thiol oxidant diamide (Jeong et al., 1999; Lee et al., 1999), we then tested the roles of *URM1* and *AHP1* in diamide stress response. Interestingly, compared to the wild-type strain, both the Δ*urm1* and Δ*ahp1* mutants were evidently sensitive to 1.5 mM diamide, and Δ*urm1* was more sensitive than Δ*ahp1* (Figure 8), indicating urmylation in *M. oryzae* should play important roles in responding to the thiol oxidant diamide. It is reported that the urmylation is also involved in the TOR signaling pathway in *S. cerevisiae*, and we also tested the sensitivity of Δ*urm1* and Δ*ahp1* to rapamycin, an inhibitor of the TOR signaling pathway. As expected, the Δ*urm1* mutant was significantly sensitive to rapamycin, while the Δ*ahp1* mutant was more resistant (Figure 8). This result indicated the urmylation pathway also plays a role in the TOR signaling pathway.

### URM1-Mediated Urmylation Is Responsible for Modification of Ahp1

**FIGURE 9:**
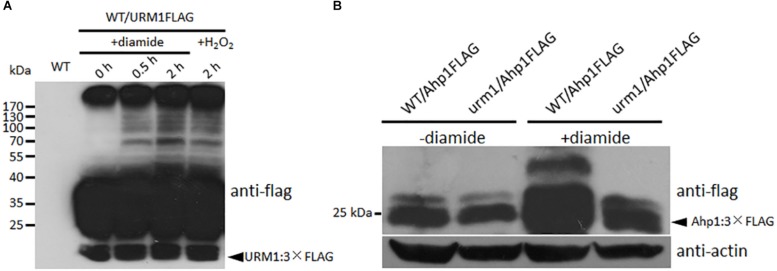
URM1 is responsible for modification of Ahp1. **(A)** Total urmylation level is responsive to oxidative stress. Total lysates of the WT/URM1FLAG strain under different conditions were subjected to SDS-PAGE and immunoblotted using anti-flag antibodies to detect total urmylation levels. **(B)** Ahp1 is modified by URM1. Ahp1:3 × Flag fusion constructs were transformed into the wild type and the Δ*urm1* mutant, respectively. Corresponding transformants cultured with or without 1.5 mM diamide were analyzed by western blotting with anti-flag antibody. Triangle indicates the Ahp1:3 × Flag fusion protein bands. The β-actin levels detected by anti-actin were used as a loading control.

To detect the effect of oxidative stresses on urmylation, the wild-type strain expressing the URM1:3 × FLAG fusion construct was incubated in CM medium containing different oxidative stresses. Total proteins of the mycelia were extracted and immuno-blotted with anti-FLAG antibody. In the control without oxidative stress, bands can seldom be detected beside the URM1:3 × FLAG protein itself, while in the oxidative conditions, many larger bands can be detected, and more bands can be detected in the 0.5 mM diamide and 2 mM H_2_O_2_ conditions (Figure 9A). This result showed the oxidative condition, especially the thiol oxidant diamide condition, can induce urmylation in *M. oryzae*.

In order to clarify whether Ahp1 is modified by URM1-mediated urmylation, the Ahp1:3 × Flag fusion construct was transformed into the wild type and the Δ*urm1* mutant, respectively. The subsequent transformants were incubated for 48 h in the CM or CM supplemented with 1.5 mM diamide. Total proteins from the samples were extracted and immuno-blotted by anti-flag antibody. In the control CM condition, no different bands could be detected between WT/Ahp1FLAG and *urm1*/Ahp1FLAG. In the oxidative conditions, a larger band could be detected in WT/Ahp1FLAG (Figure 9B), indicating a modification was occurred. Taken together, the URM1-mediated urmylation is responsible for modification of Ahp1, which is dependent on cellular oxidative stresses.

## Discussion

Post-translational modifications mediated by ubiquitin-like proteins play key roles in regulation of diverse cellular processes. Previous studies have revealed importance of the urmylation in *S. cerevisiae* and mammals. However, little has been addressed on roles of urmylation in other organisms. In this study, we firstly investigated functions of the urmylation in the plant pathogenic fungus *M. oryzae*. By functional analysis of the *M. oryzae URM1* deletion mutant, we found this modification pathway plays roles in fungal vegetative growth and conidium formation, and it is also involved in regulating virulence by affecting invasive growth in host cells. Moreover, this ubiquitin-like modification is important for cell wall integrity. Further, we found the URM1-mediated urmylation plays an important role in detoxification of host oxidative stresses. We also identified that thioredoxin Ahp1 is modified by URM1, which is dependent on diamide oxidative stress.

We reasoned that attenuation in virulence of the Δ*urm1* mutant could be due to several cellular mechanisms. The invasive growth of the Δ*urm1* mutant in host cells was evidently arrested (Figures 5D,E); this defect could most probably result from reduction in suppressing host oxidative stress. Through the DAB staining assay, we observed a massive accumulation of ROS in Δ*urm1* infected barley epidermis cells (Figure 7). The Δ*urm1* mutant was also slightly sensitive to H_2_O_2_ and significantly sensitive to diamide. The diamide can oxidize cellular thiols and induces oxidative stress in plant cells for defense response (Kosower and Kosower, 1995). In this way, the urmylation-mediated detoxification of host diamide-induced oxidative stress could be a novel mechanism for fungal infection. We further found that diamide can induce urmylation level *in vivo*, and a thioredoxin Ahp1, whose gene’s deletion mutant was sensitive to diamide-derived oxidative stress, was modified by URM1 (Figures 9A,B). It is strange that the highest expression of *URM1* occurs during the appressorium formation stages, while the Δ*urm1* mutant is normal in appressorium formation but arrested in invasive growth. We infer that URM1-mediated modification of Ahp1 may be started during appressorium formation, and the effect may not reflect in appressorium formation ratio, but a preparation for invasive growth.

When *M. oryzae* infects on susceptible host plant, it can penetrate into the host cell and form IH for invasive growth and then colonize adjacent cells through plasmodesmata (Kankanala et al., 2007). When the fungus infects a resistant host plant, it cannot penetrate the host cell due to the host hypersensitive response (HR) and programed cell death (Heller and Tudzynski, 2011). Plant host can generate pathogen-associated molecular pattern (PAMP)-triggered immunity, known as PTI, or effector-triggered immunity, known as ETI, for defense response (Thomma et al., 2011). During both PTI and ETI, rapid accumulation of ROS and other oxidative stress is a key process for blocking IH expansion (Tudzynski et al., 2012). On the other hand, *M. oryzae* also developed effective detoxification systems to counteract host oxidative stress for infection. Many anti-oxidant genes exist in the *M. oryzae* genome (Egan and Talbot, 2008; Morel et al., 2008), and several of them are known, including genes encoding glutathione peroxidase Hyr1 (Huang et al., 2011), redox-sensitive transcription factors MoAP1 (Guo et al., 2011) and MoAtf1 (Guo et al., 2010), defense suppressor protein Des1 (Chi et al., 2009), glutathione reductase (GTR1), thioredoxin reductase (TRR1), thioredoxin peroxidase (TPX1) (Fernandez and Wilson, 2014) and peroxidase MoPRX1 (Mir et al., 2015). In this study, we identified URM1 as another component of the detoxification systems, which is used to counteract host oxidative stress for infection of *M. oryzae*.

On the other hand, the Δ*urm1* mutant was also sensitive to rapamycin (Figure 8), indicating the urmylation pathway should be linked to the TOR signaling pathway in *M. oryzae*. As the TOR signaling pathway has been proved to play key roles in nutrient assimilation during invasive growth of *M. oryzae*, and the Δ*urm1* mutant is also defect in growth on the nutrient oatmeal agar plate, we infer that the arresting of invasive growth for the mutant could also be related to affecting of this pathway. However, the relationship between urmylation and the TOR signaling pathway is still unclear. Recent studies suggest the TOR signaling pathway may also be involved in oxidative stress response (Weisman and Choder, 2001). It is also reported that URM1 can also act as a sulpur carrier in thiolation of eukaryotic transfer RNA (tRNA) to regulate cellular responses to nutrient starvation and oxidative stress conditions (Leidel et al., 2009; Jüdes et al., 2016; Termathe and Leidel, 2018). URM1-mediated wobble uridine modification of tRNA is also reported to be required for proper TOR signaling (Scheidt et al., 2014). Therefore, it is interesting to determine whether URM1 is required for tRNA thiolation and TOR signaling during invasive growth of *M. oryzae*.

URM1 has been firstly identified in *S. cerevisiae* and was found to be important for budding and growth at high temperatures (Furukawa et al., 2000; Goehring et al., 2003a). URM1 was also found in HeLa cells, in which it plays roles in cytokinesis (Schlieker et al., 2008) and cellular defense against oxidative stress (Goehring et al., 2003a, b; van der Veen et al., 2011). Since then, in yeast, mammalian cells and *Drosophila melanogaster*, it was found that the oxidative stress can evidently increase cellular urmylation levels (Goehring et al., 2003a; van der Veen et al., 2011; Khoshnood et al., 2016). These studies suggest an evident linkage of urmylation and oxidative stress, which is consistent with our study. Besides Ahp1, recent proteomic studies have identified numerous targets of urmylation, including in mammalian cells (van der Veen et al., 2011), *Haloferax volcanii* (Humbard et al., 2010) and Drosophila (Judes et al., 2015; Khoshnood et al., 2016); most of them were also involved in oxidative stress detoxification. In the future, it will also be necessary to use proteomic tools to identify targets of urmylation in fungi, which could help us to further reveal the regulatory mechanisms of this ubiquitin-like modification.

## Data Availability

The raw data supporting the conclusions of this manuscript will be made available by the authors, without undue reservation, to any qualified researcher.

## Author Contributions

LW, XC, CL and AH performed most of the experiments and data processing. XC and JX designed the experiments. XC and JX wrote the manuscript.

## Conflict of Interest Statement

The authors declare that the research was conducted in the absence of any commercial or financial relationships that could be construed as a potential conflict of interest.

## Footnotes

1 http://fungi.ensembl.org/Magnaporthe_oryzae/Info/Index

## Supplementary Material

FIGURE S1 Phase specific expression of *URM1*.

FIGURE S2 Replacement of *URM1* in *M. oryzae*.

FIGURE S3 Appressorium formation rates of the wild type, Δ*urm1* mutant and complemented strains.

TABLE S1 Strains used in this study.

TABLE S2 Vectors used in this study.

TABLE S3 Primers used in this study.
