# Supplementary material for: URM1-Mediated Ubiquitin-Like Modification Is Required for Oxidative Stress Adaptation During Infection of the Rice Blast Fungus
Source: Front Microbiol. 2019 Sep 10;10:2039. doi: 10.3389/fmicb.2019.02039 (PMC6746893; doi:10.3389/fmicb.2019.02039)
Supplement: FIGURE S1 — Phase specific expression of URM1. [file Data_Sheet_1.PDF]

**Supporting data Figs S1–S1, Table S1**

Urm1 mediated ubiquitin-like modification is required for oxidative stress adaptation during infection of the rice blast fungus

Authors: Luyang Wang[#], Xuan Cai[#], Junjie Xing, Caiyun Liu, Ahmed Hendy, Xiao-Lin Chen*

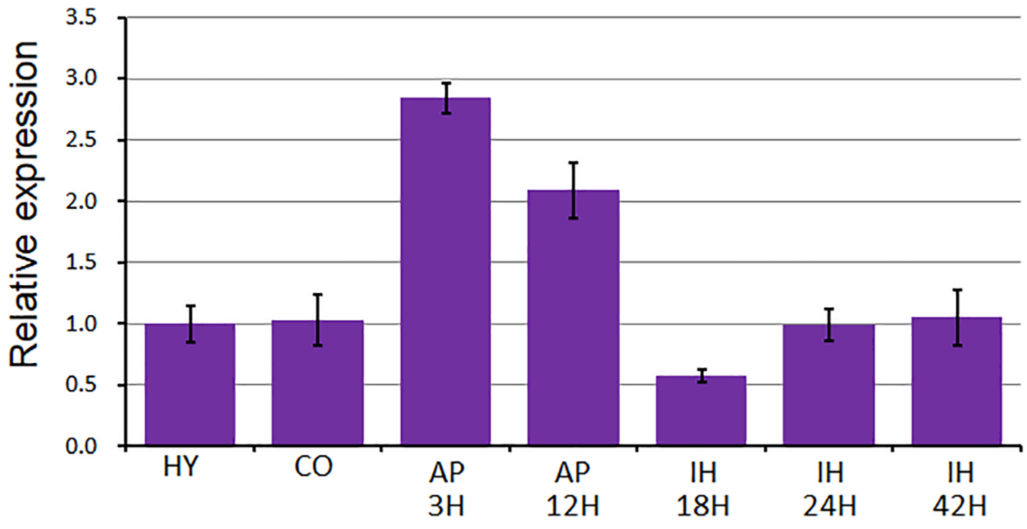


**Figure S1 |** Phase specific expression of *URM1*. The phase specific expression of *URM1* was quantified by quantitative real-time PCR with synthesis of cDNA from each sample including mycelia, conidia, germ tubes, appressoria and infection hyphae at indicated time points. Relative abundance was normalized by *MoTub1*. The expression level of *URM1* in mycelium was set as 1, expression of other stages were relative to the mycelium stage. We have clarified this in the method section. Means and standard errors were calculated from three independent replicates. HY : Mycelial hyphae; CO: Conidia; AP : Appressoria ; IH : infection hyphae.

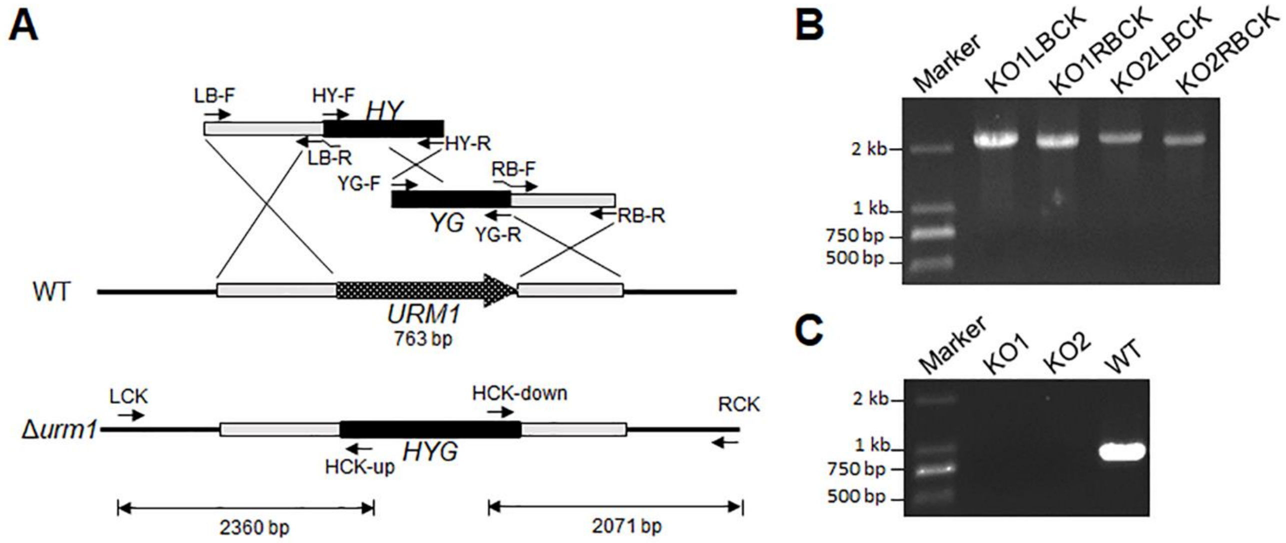


**Figure S2 |** Replacement of *URM1* in *M. oryzae*. (A) Gene replacement of *URM1* through a split-marker approach. White bars represent genomic regions upstream and downstream of the *URM1* coding sequence that were amplified and fused to segments of the hygromycin phosphotransferase (HYG) cassette. (B) PCR verification of the flanking sequences beside the replacement fragment by using primer pairs of LCK/HCK-up and RCK/HCK-down. (C) RT-PCR verification by amplifying the expression of the *URM1* gene in the transformants and the wild-type strain.

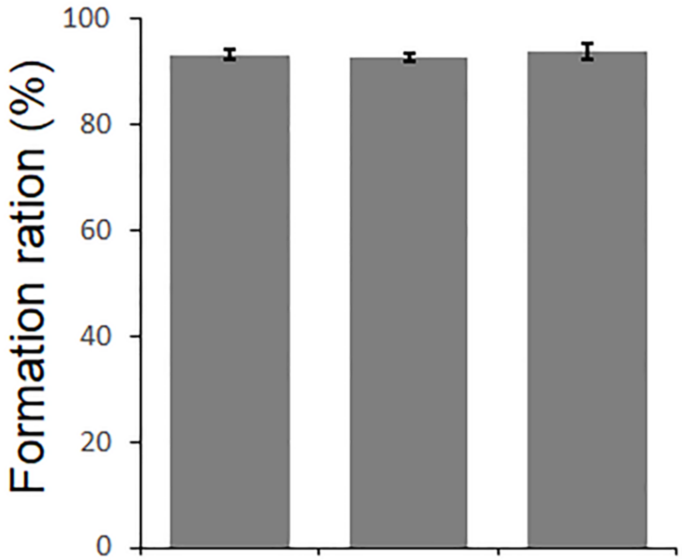


**Figure S3 |** Appressorium formation rates of the wild type, Δ*urm1* mutant and complemented strains. Conidia were incubated on hydrophobicsurfaces and observed under a microscope at 12 h.

**Table S1 Fungal strains used in this study.**

| Strains | Genotypes | References |
|---|---|---|
| P131 | A wild-type isolate of *M. oryzae* | (Peng and Shishiyama, 1988) |
| KO1, KO2 | *URM1* deletion mutants of P131, Δ*urm1*. | This study |
| cURM1 | Complement strain of Δ*urm1*, Δ*urm1/URM1* | This study |
| URM1G | P131 transformed by *eGFP-URM1* fusion construct, WT/GFP:Urm1. | This study |
| WT/URM1FLAG | P131 transformed by *URM1:3×Flag* fusion construct, WT/GFP:Urm1. | This study |
| WT/Ahp1FLAG | P131 transformed by *AHP1:3×Flag* fusion construct. | This study |
| urm1/Ahp1FLAG | Δ*urm1* transformed by *AHP1:3×Flag* fusion construct. | This study |

**Table S2 Plasmids used in this study.**

| **Names** | **Descriptions** |
|---|---|
| pKN | Vector used to construct complementation vectors and other vectors; with the *NPTII* gene as a selective marker inserted into $pKS^{+}$ (Yang et al., 2010). |
| pKN-*URM1* | *URM1* complementation vector; *URM1* gene containing 1.5 kb promoter and 0.5 kb terminator regions were amplified and inserted into pKN. |
| pKNRG | Vector used to construct vectors to constitutively express selected genes; with the fungal constitutive promoter RP27 (Yang et al., 2010). |
| pKNRG-*URM1* | Vector for sub-cellular localization of Urm1 protein; coding region of *URM1* was cloned into vector pKNRG. |
| pKNFLAG | Vector used to construct vectors expressing 3xFLAG fusing protein promoted by constitutive promoter RP27. |
| pKNFLAG-*URM1* | Vector constitutively expressing URM1:3×FLAG. |
| pKNFLAG-*AHP1* | Vector constitutively expressing AHP1:3×FLAG. |

**Table S3 Plasmids used in this study.**

| Primers | Sequences (5'–3') |
|---|---|
| HPT-LCK | GACAGACGTCGCGGTGAGTT |
| HPT-RCK | TCTGGACCGATGGCTGTGTAG |
| HPT-F1 | CTCCGACCTGATGCAGCTCT |
| HPT-R1 | CTCGCTCCAGTCAATGACC |
| HYG-LB | ACCTCCACTAGCTCCAGCCAAG |
| HYG-RB | GAATAGAGTAGATGCCGACCGGG |
| URM1LBCK | GGCTGGCCAAGGATCTGTAG |
| URM1LB-F | GGTGAGCACTTCTGTAGTTGG |
| URM1LB-R | ACCTCCACTAGCTCCAGCCAAGGACAACGGTGCCCATTGC |
| URM1RB-F | GAATAGAGTAGATGCCGACCGGGAGTGAGGTTATGCCGAGA |
| URM1RB-R | TTGATTCACTCTCCCACTCC |
| URM1RBCK | ACTGGGATTCGCTAGCCTCG |
| AHP1LBCK | CAACCTCGATGCCATCTC |
| AHP1LB-F | GACTGCATCCACGCGTAT |
| AHP1LB-R | ACCTCCACTAGCTCCAGCCAAGAGGTGGGATAAATGCTCC |
| AHP1RB-F | GAATAGAGTAGATGCCGACCGGGCCCGATTCGTGTTCGTCT |
| AHP1RB-R | CGAGTCACTGATGCTGCA |
| AHP1RBCK | GGATGACTTGTAGTTGCGGAC |
| cURM1-F | GTCGACGGTATCGATAAGCTTTACTTGTCCTGACTCGCAGAG |
| cURM1-R | CCCGAATTCGATATCAAGCTTCTAGCCTCCATGCAGAGTC |
| URM1GFP-F | TTGAAGCTTATGCACCAGGTTGAGACAGT |
| URM1GFP-R | GTTGGATCCCTAGCCTCCATGCAGAGTCG |
| AHP1FLAG-F | AAACCCGGGCTGCAGGAATTCATGGCATTCAGAGCATTTAG |
| AHP1FLAG-R | GATAAGCTTGATATCGAATTCTCCCAGAACCTTGTCGGC |
| URM1qF | GTTGGGGATGACGACATG |
| URM1qR | AGAATCCATCTCTTGAAG |
